# The Treatment of Healthy Ulcers of the Extremities by “Sealing”

**Published:** 1864-08

**Authors:** W. B. Slayter

**Affiliations:** Chicago, Graduate Royal College of Physicians of England, Royal College of Surgeons of London and Dublin. Late House-Surgeon of Westminster Hospital, London


THE TREATMENT OF HEALTHY

### ULCERS OF THE EXTREMITIES BY "SEALING."

By W. B. SLAYTER, M. D., of Chicago,

*Graduate Royal College of Physicians of England, Royal College of Surgeons of London and Dublin. Late House-Surgeon of Westminster Hospital, London.*

The treatment of healthy ulcers of the extremities by sealing, has received little or no attention from the profession in this part of the country, although in many of the Eastern cities and Europe, it is employed by many of the most eminent surgeons.

The treatment consists in applying around the ulcer and in the sound skin, narrow strips of adhesive plaster. A piece of thin oil silk is then placed over the ulcer and affixed to the plaster by means of collodion; the part is then bandaged and kept as quiet as possible. The advantages of this treatment are, 1st. Healthy ulcers heal more rapidly than by the ordinary methods of treatment. 2d. It gives very much less trouble. 3d. The surface of the ulcer can always be seen without removing the dressings.

1st. Numerous cases have been published in "Braithwait's Retrospect," and other journals, clearly showing that ulcers which are inclined to heal slowly, have, under this treatment, very rapidly put on a healthy appearance and filled up; and in my own practice at the Westminster Hospital and other places have witnessed the marked success which has attended its use. In the Westminster Hospital, where numerous cases are always to be found, the various treatments have been tested, and in the majority sealing has been found most beneficial; so much so in fact that in most of the London Hospitals it is employed as a local dressing almost altogether. It prevents access of the air to the surface of the ulcer, thereby assisting materially in rapid cicatrization.

2d. It gives far less trouble both to the surgeon and patient, as it requires to be removed only once in three or four days, or where there is little suppuration, once in a week or ten days. Every surgeon knows the importance of leaving an ulcer undisturbed as long as possible, so that the new skin as it forms may not be pulled off with the dressings. Independent of these advantages this treatment in some cases very materially relieves the dull aching pain attending some forms of ulcers.

In conclusion, I would strongly recommend it to the notice of the profession, as I am convinced that in the majority of cases they will be well pleased with it, and inclined to use it to the exclusion of all other local dressings.

